# Der p1 Dendritic Cells Promote Regulatory B Cell Induced Immunotolerance Through IL-10/STAT3 in Allergic Rhinitis

**DOI:** 10.3390/biomedicines14010206

**Published:** 2026-01-18

**Authors:** Kai Fan, Ling Jin, Chuanliang Zhao, Shican Zhou, Shiwang Tan, Ju Lai, Chunyan Yao, Bojin Long, Yawen Gao, Shaoqing Yu

**Affiliations:** 1 Department of Otorhinolaryngology-Head and Neck Surgery, Tongji Hospital, School of Medicine, Tongji University, 389 Xincun Road, Putuo District, Shanghai 200065, China; 2 Department of Allergy, Tongji Hospital, School of Medicine, Tongji University, Shanghai 200065, China

**Keywords:** allergens, B-lymphocytes regulatory, interleukin-10, rhinitis allergic

## Abstract

**Background/Objectives**: Allergic rhinitis (AR) is a complex immune-mediated disorder characterized by defective regulatory mechanisms. Emerging evidence suggests that impaired immune tolerance mediated by regulatory B cell (Breg) plays a pivotal role in AR pathogenesis. This study investigates the therapeutic potential of Der p1 allergen-modified dendritic cells (DC) in enhancing Breg-mediated immunotherapy and explores novel mechanisms underlying AR immunomodulation. **Methods**: Breg and the inflammatory cytokines were detected before and after allergen immunotherapy (AIT) in AR patients. Dust mite gene-derived dendritic cells were used to induce Breg. AR mice were treated with Der p1-DCs, and changes in Breg and related inflammatory indicators, as well as the impact of the IL-10/STAT pathway on DC vaccine treatment, were observed. **Results**: Following 6-month AIT, AR patients exhibited significant alleviation of nasal symptoms alongside restored peripheral Breg and Treg. In vitro co-culture of Der p1-DC-induced Bregs with CD4^+^CD25^−^T cells revealed that IL-10 blockade markedly increased Th cell. In AR murine models, intraperitoneal Der p1-DC administration suppressed allergic symptoms, upregulated nasal mucosal IL-10 expression, and attenuated STAT3 phosphorylation via IL-10 overexpression. **Conclusions**: AIT establishes immune tolerance through Breg-mediated regulatory mechanisms, while Der p1-DCs potently induce Breg differentiation and drive tolerance induction via the IL-10/STAT3 signaling axis.

## 1. Introduction

Allergic rhinitis (AR) is a non-infectious chronic inflammatory disorder of the nasal mucosa that is mediated by immunoglobulin E (IgE) following the exposure of an atopic individual to allergens [1]. Owing to the disparities among global regions and the multiplicity of terrains, climates, and climatic conditions, allergens exhibit marked differences across different areas. In warm regions, the sensitization rate of dust mites is the highest, whereas plants like mugwort and dandelion are the predominant allergens in northern regions [2]. AR has been designated as the most prevalent chronic respiratory inflammatory disease by the Global Burden of Disease Study Center. Over the past several decades, AR has affected billions of people worldwide [3,4]. Moreover, due to its recurrent nature and the diversity of allergens, AR has imposed a colossal economic burden on the medical and social sectors. Consequently, it is of utmost significance to investigate the pathogenesis of AR and formulate novel treatment strategies.

The theory that is more widely acknowledged within the current domain posits that AR is a hypersensitivity disease resulting from immunological tolerance deficiency [5,6]. Immunological tolerance, serving as a crucial means for the body to uphold immune equilibrium, principally down-regulates the individual immune response via the immune system, thereby diminishing or even averting the uncontrolled lymphocyte proliferation or abnormal immune responses upon antigenic stimulation. Hence, the induction and maintenance of immunological tolerance represent a vital mechanism for ensuring the body’s immune balance and preventing allergic inflammation [5].

Symptomatic alleviation of AR is attained via pharmacological therapies and, in refractory cases, established surgical interventions—which can be broadly categorized into structural surgeries (e.g., turbinate reduction or septoplasty) to improve nasal airflow and address obstruction, and neural blockade procedures (e.g., vidian neurectomy) to reduce mucosal reactivity and control rhinorrhea, sneezing, and itching—while the causal treatment of AR is effected through allergen-specific immunotherapy (AIT) [7]. AIT is presently a frontline therapeutic modality endorsed in clinical practice. Its objective is to augment the dosage of sensitizing allergens by means of subcutaneous immunotherapy or sublingual immunotherapy, with the intention of inducing immunological tolerance towards allergens such as pollen, dust mites, or molds [8]. Research has unveiled that AIT exerts its inhibitory effect on allergic reactions through the collaborative action of a constellation of immune cells and a gamut of anti-inflammatory and pro-inflammatory cytokines. This entails the attenuation of mast cell and basophil activity, an increment in the numbers of peripheral and local regulatory B cells (Breg) and regulatory T cells (Treg), and an elevation in the levels of allergen-specific IgG4 [9,10]. Previous investigations have established that regulatory cells like Treg and associated inflammatory mediators are pivotal elements in upholding the body’s immune homeostasis in AR [11,12]. More recent studies have also spotlighted a particular subset of B cells, namely IL-10^+^Breg, which is distinguished by its capacity to secrete IL-10 and modulate the body’s immune responses. The regulatory function of IL-10^+^Breg in allergen tolerance is now acknowledged as a significant immunological tolerance mechanism in AR [13].

Despite AIT demonstrating a satisfactory therapeutic outcome, its clinical application is circumscribed owing to the low efficacy of the immune response, which consequently gives rise to a protracted treatment cycle. In the event that the gene-modified immune cell technology is harnessed, the Dermatophagoides pteronyssinus 1 (Der p1) allergen gene is transfected into dendritic cells (DCs) in vitro to fabricate a DC vaccine (Der p1-DC), and subsequently introduced into the body to effectively prompt Breg to augment immunological tolerance, it ought to be capable of more effectively enhancing the immunotherapy efficiency of AR. Grounded on this premise, this study endeavors to dissect the mechanism of Breg-mediated immunological tolerance elicited by clinical immunotherapy in AR patients and to scrutinize the augmentation of Breg-mediated immunological tolerance by the Der p1-DC vaccine via in vitro and animal experiments.

## 2. Materials and Methods

### 2.1. Study Subjects

The study subjects were randomly selected from 30 patients diagnosed with allergic rhinitis (AR) according to the “Allergic rhinitis and its impact on asthma” who visited our outpatient clinic between October 2020 and December 2022. All subjects participated voluntarily and signed informed consent forms. All patients underwent subcutaneous injection therapy with Alutard SQ (house dust mite allergen extract) (ALK-Abello A/S, Horsholm, Denmark). To assess the early efficacy of allergen immunotherapy, total nasal symptom score and visual analog scale were evaluated at baseline and after 6 months of treatment, which served as a pre-specified mid-term evaluation point within the course of therapy.

### 2.2. Total Nasal Symptom Score (TNSS)

All subjects were evaluated for nasal symptoms using the standardized TNSS system, which assesses four core nasal symptoms: nasal congestion, rhinorrhea, sneezing, and nasal itching. Each symptom was graded on a 4-point scale (0–3 points) based on severity, with scores summed to obtain the total TNSS.

The specific grading criteria were as follows: 0 points = no symptoms; 1 point = mild symptoms (symptoms present but not interfering with daily activities or sleep); 2 points = moderate symptoms (symptoms noticeable and occasionally interfering with daily activities or sleep); 3 points = severe symptoms (symptoms persistent and significantly interfering with daily activities, work, or sleep).

### 2.3. Visual Analog Scale (VAS)

The VAS was used to assess the subjective severity of overall nasal discomfort in human participants. A horizontal straight line of 10 cm in length was used, with the left end (0 cm) representing “no nasal discomfort at all” and the right end (10 cm) representing “the most severe nasal discomfort imaginable.”

Participants were instructed to mark a point on the line corresponding to their actual discomfort level independently. The distance from the left end (0 cm) to the marked point was measured with a ruler to obtain the VAS score (unit: cm), with higher scores indicating more severe subjective discomfort.

### 2.4. ELISA Detection of IL-10 and TGF-β Levels in Human Serum

Peripheral venous blood from AR patients and healthy individuals was collected into separator gel-promoted coagulation tubes, shaken uniformly, and allowed to stand at room temperature for 2 h. After blood coagulation, it was centrifuged at 1000× *g* for 10 min using a low-speed centrifuge. The upper yellow liquid, i.e., serum, was transferred to a 1.5 mL EP tube and stored at −20 °C. Enzyme-linked immunosorbent assay (ELISA) kits (Shanghai Lianshuo Biological Technology Co., Ltd., Shanghai, China) were used to detect human serum IL-10 and TGF-β. Each sample was analyzed in triplicate, and the average value was calculated.

### 2.5. ELISA Detection of Specific IgE Levels in Mouse Serum

Peripheral blood of mice was collected and allowed to stand undisturbed in EP tubes at room temperature for 1 h. Subsequently, the samples were centrifuged at 1000× *g* for 10 min, and the supernatant was harvested, which was designated as serum and then stored at −80 °C. ELISA kits (Shanghai Lianshuo Biological Technology Co., Ltd., Shanghai, China) were used to detect mouse serum specific IgE. Each sample was analyzed in triplicate, and the average value was calculated.

### 2.6. Flow Cytometry Detection of Breg, Treg, Th1, Th2, and Th17

Human Peripheral blood mononuclear cells (PBMCs) were isolated as follows: 2 mL fresh peripheral blood was collected into heparinized tubes and diluted with equal normal saline. 2 mL lymphocyte separation medium was added to a 15 mL tube, with 4 mL diluted blood layered gently onto it for a clear interface. The sample was centrifuged at 800× *g* for 20 min at room temperature (horizontal rotor). After centrifugation, four layers formed: plasma, buffy coat (mononuclear cells), separation medium, and red blood cells. The buffy coat was aspirated into a new tube, diluted, mixed by inversion, centrifuged at 250× *g* for 10 min, and washed 1–2 times after discarding the supernatant. Finally, PBMCs were resuspended in PBS or culture medium for use. Briefly, PBMCs were stained with anti-CD4-PE, anti-CD25-FITC, and anti-CD127-PC5 monoclonal antibodies to identify human Tregs, anti-CD19-PE, anti-CD24-PC5, and anti-CD38-FITC antibodies to identify human Bregs (Beckman Coulter, Brea, CA, USA), and anti-CD4-FITC, anti-IFN-γ-BV421, and anti-IL-4-PE (Biolegend, San Diego, CA, USA) to identify Th1 and Th2. Mouse Cell was label with antibodies CD19-FITC, CD1d-PE, and CD5-APC (Biolegend, USA) for mouse Breg; and CD4-FITC, IFN-γ-BV421, IL-4-PE, and IL-17A-APC (Biolegend, USA) for mouse Th1, Th2, and Th17. To detect intracellular cytokines, Cell activation Cocktail (5 μg/mL, Biolegend) was added to stimulate the cells 5 h before the end of the culture. Data were acquired on a FACSCalibur (Beckman Coulter, USA) and analyzed using FlowJo V.10.1 software (BD, Franklin Lakes, NJ, USA).

### 2.7. Culture of Mouse Bone Marrow-Derived Dendritic Cells (BMDC)

Six- to ten-week-old C57 mice were euthanized by cervical dislocation and immersed in 70% alcohol for 5 min. The femurs and tibias of the mice were aseptically removed, and the ends of the bones were cut off with scissors. RPMI 1640 was drawn into a 1 ml syringe, and the needle was inserted into the bone marrow cavity from both ends to repeatedly flush out the bone marrow and collect bone marrow cells. The bone marrow cells were plated in a 6-well culture plate and simultaneously added with recombinant mouse GM-CSF (20 ng/mL, PeproTech, Cranbury, NJ, USA) and IL-4 (10 ng/mL, PeproTech, USA) for culture in a 37 °C, 5% CO_2_ incubator. On day 4, recombinant mouse GM-CSF (20 ng/mL) and IL-4 (10 ng/mL) were supplemented into the culture dish. On day 6 of culture, DCs were collected, which were immature BMDCs (mDCs).

### 2.8. Preparation of Der p1-DC Vaccine

Based on the sequence published in GenBank, the target sequence Der p1 (GenBank Accession No.: EU092644.1) was synthesized in full length, and the pCDNA3.1(+)-Der p1-GFP plasmid vector was designed and synthesized. It was then ligated with the pLenti6.3-MCS/V5 DEST lentiviral expression vector using a ligase to form the lentiviral expression vector Der p1-GFP. Mouse mDC cells were collected in 1.5 mL Ep tubes from, and virus solution was added according to an MOI of 100:1. The transfected cells were observed after 48 h to obtain the Der p1-DC vaccine.

### 2.9. Experiment on Breg Induction by DC Vaccines in B Cells

Magnetic bead separation was used to isolate CD19^+^B cells from the spleens of AR mice, which were then cultured in a 24-well plate. A total of 10 μg/mL LPS (InvivoGen, San Diego, CA, USA) was added to stimulate Breg differentiation. After incubation in a 37 °C, 5% CO_2_ incubator for 48 h, the cells were collected. The collected LPS-B cells were co-cultured with DC vaccines, and the expression level of IL-10 in the supernatant was detected after 48 h. Flow cytometry was used to detect the levels of CD19^+^CD5^+^CD1d^+^Breg.

### 2.10. Experiment on the Induction and Differentiation of Th by Breg

The 96-well U-bottom culture plates were coated with CD3 antibody and CD28 antibody (both at 10 μg/mL, Biolegend). Flow cytometry was used to sort CD19^+^CD5^+^CD1d^+^ Breg cells, which were then co-cultured with CD4^+^CD25^-^T cells at a 1:1 ratio in 96-well plates, with or without αIL-10 (5 μg/mL, Biolegend, USA) and/or TGF-β inhibitor SB431542 (10 μmol/L, Peprotech, USA) for 72 h. The effects of IL-10 and TGF-β secreted by Breg on T cell differentiation were observed.

### 2.11. RT-PCR Detection of Cytokine and Transcription Factor mRNA

Total RNA was extracted from nasal mucosa with Trizol (Invitrogen, Carlsbad, CA, USA). Briefly, tissue in 1 mL Trizol (EP tube) with a stainless steel bead was lysed homogenously (Qiagen, Germantown, MD, USA), then centrifuged at 12,000× *g* for 15 min (RT) to remove debris. Supernatant was transferred to a new tube, mixed with 200 μL chloroform (vortexed), incubated 5 min (RT) for phase separation, centrifuged at 12,000× *g* for 15 min (4 °C), and upper RNA phase collected. Equal-volume isopropanol was added (inverted gently), incubated 5 min (RT) to precipitate RNA. After centrifugation (12,000× *g*, 15 min, 4 °C), supernatant was discarded. RNA pellet was washed with 1 mL 75% ethanol, centrifuged (12,000× *g*, 5 min, 4 °C), and supernatant removed. Pellet was washed again with 1 mL anhydrous ethanol (same centrifugation), air-dried 5 min (RT), resuspended in 20 μL DEPC water, and stored at −80 °C for qRT-PCR.

Total RNA extracted from mouse nasal mucosa or cells reverse transcribed into cDNA, and amplified for fluorescence detection. The PCR reaction conditions were as follows: 95 °C for 1 min; (95 °C for 20 s, 60 °C for 1 min) × 40 cycles. Relative gene expression was analyzed using the 2^−ΔΔCt^ method. GAPDH was used as the internal reference gene, and t-Bet, GATA3, RORγt, IL-10, IL-4, and IFN-γ were used as the target genes. The specific primer sequences were as follows:

- GAPDH: 5′-CCCTTAAGAGGGATGCTGCC-3′, 5′-TACGGCCAAATCCGTTCACA-3′;
- Tbet: 5′-ATTGGTTGGAGAGGAAGCGG-3′; 5′-GCACCAGGTTCGTGACTGTA-3′;
- GATA3: 5′-TGGCGCCGTCTTGATAGTTT-3′; 5′-GCCCGGTCAGATTGCGTA-3′;
- RORγt: 5′-TCTTTAACTCCCTTGGCGCA-3′; 5′-TCAGGGTCTTCATTGCGGTG-3′;
- IL-10: 5′-AGGCGCTGTCATCGATTTCT-3′, 5′-TGTTACACTCGCCCCCTTTG-3′;
- IL-4: 5′-CCATATCCACGGATGCGACA-3′, 5′-CGTTGCTGTGAGGACGTTTG-3′;
- IFN-γ: 5′-AGCAAGGCGAAAAAGGATGC-3′, 5,-TCATTGAATGCTTGGCGCTG-3′

### 2.12. Model Construction and Immunotherapy of AR Mice

Using block randomization, the twenty-four 6–8-week-old C57 mice, each weighing around 20–30 g, were equally distributed into four groups (*n* = 6 per group): Control, AR, Der p1-DC treatment group (Der p1/AR group), and IL-10 antibody blockade group (αIL-10/AR group). The mice were housed in a Specific Pathogen-Free (SPF) animal laboratory. The SPF facility implements strict microbial control, and the experimental environment rigorously regulates the entry and exit of personnel, materials, and air. Mice in the AR group and immunotherapy groups were intraperitoneally injected with 10 μg of Der p1 extract (GreerLabs, Lenoir, NC, USA) on days 0, 7, and 14. From day 15, mice were intranasally instilled with Der p1 for 7 consecutive days to induce AR, and nasal rubbing and sneezing were observed. From day 22, mice in the Der p1/AR group were intraperitoneally injected with 5 × 10^6^ units Der p-DC twice a week for 28 days, while the other groups received PBS instead. At the conclusion of the experiment, all mice were humanely euthanized. Specifically, a lethal overdose of sodium pentobarbital (150 mg/kg) was administered via intraperitoneal injection to induce deep surgical anesthesia (as indicated by the loss of toe-pinch reflex). Cervical dislocation was then immediately performed as a secondary physical method to ensure death. All mice were included in the statistical analysis.

### 2.13. HE Staining of Mouse Nasal Mucosa

Mouse nasal mucosa was isolated and fixed in 4% PFA at 4 °C overnight. After dehydration, embedding, and sectioning, the sections were stained with hematoxylin and eosin. They were then washed with 75% alcohol and anhydrous ethanol, dried, and mounted with neutral balsam. The slides were dried in a fume hood overnight.

### 2.14. Western Blot Detection of p-STAT3 and STAT3 in Mouse Spleen

Mouse spleen tissues were excised and minced into fine pieces. Western blot/IP cell lysis buffer was prepared with phenylmethanesulfonyl fluoride (PMSF, 1 mM final concentration) added minutes before use, plus a protease/phosphatase inhibitor mixture (1:100, *v*/*v*). The buffer was added at 200 μL per 20 mg tissue, and the mixture was homogenized fully. After lysis, samples were centrifuged at 12,000× *g* for 5 min at 4 °C; the supernatant was collected and stored at −20 °C. The samples were subjected to SDS-PAGE electrophoresis, membrane transfer, and blocking. The transfer membrane was then soaked in diluted primary antibody solutions for p-STAT3 and STAT3 (Hangzhou UpingBio Technology Co., Ltd., Hangzhou, China) at 4 °C overnight. After washing, the membrane was incubated with diluted secondary antibody solutions for p-STAT3 and STAT3 at room temperature for 2 h. Finally, the membrane was developed using a hypersensitive ECL chemiluminescence kit and exposed to a machine to obtain the results. The exposed results were imported into the SWE Image Gray Analysis Software (Version 3.2.1; Saiwei Biotechnology, Nanjing, China). Images with optimal exposure were selected and saved. The grayscale values of the target bands were analyzed quantitatively via the image processing system of ImageJ (Version 1.54f; National Institutes of Health, Bethesda, MD, USA).

### 2.15. Statistical Analysis

Statistical analyses of clinical and experimental data were performed using SAS 9.4 software, and graphs were generated with GraphPad Prism 8. Quantitative data are presented as mean ± standard deviation (SD), while qualitative or ordinal data are summarized as number of count and percentage. For normally distributed data, paired or unpaired *t*-tests were used to assess between-group differences. For non-normally distributed data, the Wilcoxon signed-rank test or the Mann–Whitney U test was applied. Comparisons among three or more groups were conducted using one-way ANOVA for normally distributed data and the Kruskal–Wallis test for non-normal data. A *p*-value < 0.05 was considered statistically significant for between-group comparisons. For within-group multiple comparisons, the Bonferroni correction was employed to adjust the significance level (alpha) to control type I error; here, statistical significance was defined as *p* < 0.05/m, where m represents the number of comparisons performed. Correlation analysis was carried out using the Pearson correlation coefficient.

## 3. Results

### 3.1. Demographic Characteristics

This study screened and enrolled a total of 30 patients with allergic rhinitis (AR) and 30 healthy subjects. The demographic baseline characteristics of the subjects in each group are detailed in Table 1.

### 3.2. Symptoms of AR Patients Before and After AIT

**Figure 1:**
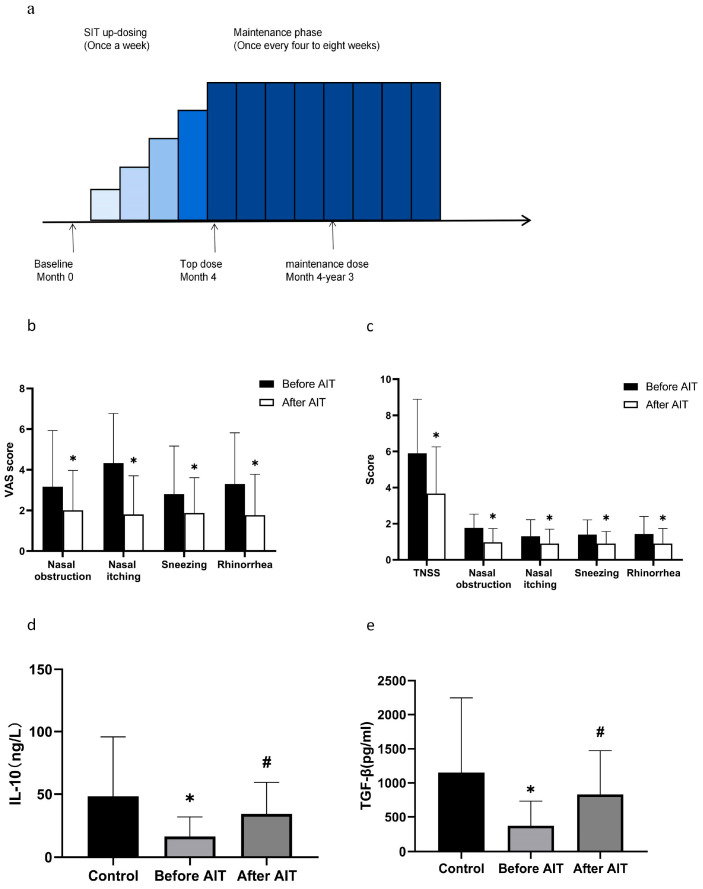
Comparison of AR patients before and after AIT treatment. (**a**) Treatment plan for AIT. (**b**) TNSS in patients with AR. The nasal symptoms of the patients were significantly improved compared with those before treatment. (**c**) VAS score of nasal symptoms in patients with AR. The nasal symptoms of the patients were significantly improved compared with those before treatment. (**d**) Comparison of serum IL-10 between healthy controls and patients before and after treatment. The level of IL-10 in AR patients increased significantly after AIT. (**e**) Comparison of serum TGF-β between healthy controls and patients before and after treatment. The level of TGF-β in AR patients increased significantly after AIT. *: Significantly different from the control group (*p* < 0.05, respectively); #: Significantly different from the before AIT group (*p* < 0.05, respectively).

The AIT process is shown in Figure 1a. After six months of AIT, AR patients exhibited significant improvement in nasal symptoms, with a notable reduction in the TNSS compared to pre-treatment levels (Figure 1b). The VAS scores also decreased significantly after treatment, with relief observed in nasal congestion, nasal itching, sneezing, and runny nose (Figure 1c).

### 3.3. Levels of Breg, Treg, and Serum IL-10, TGF-β Before and After AIT

**Figure 2:**
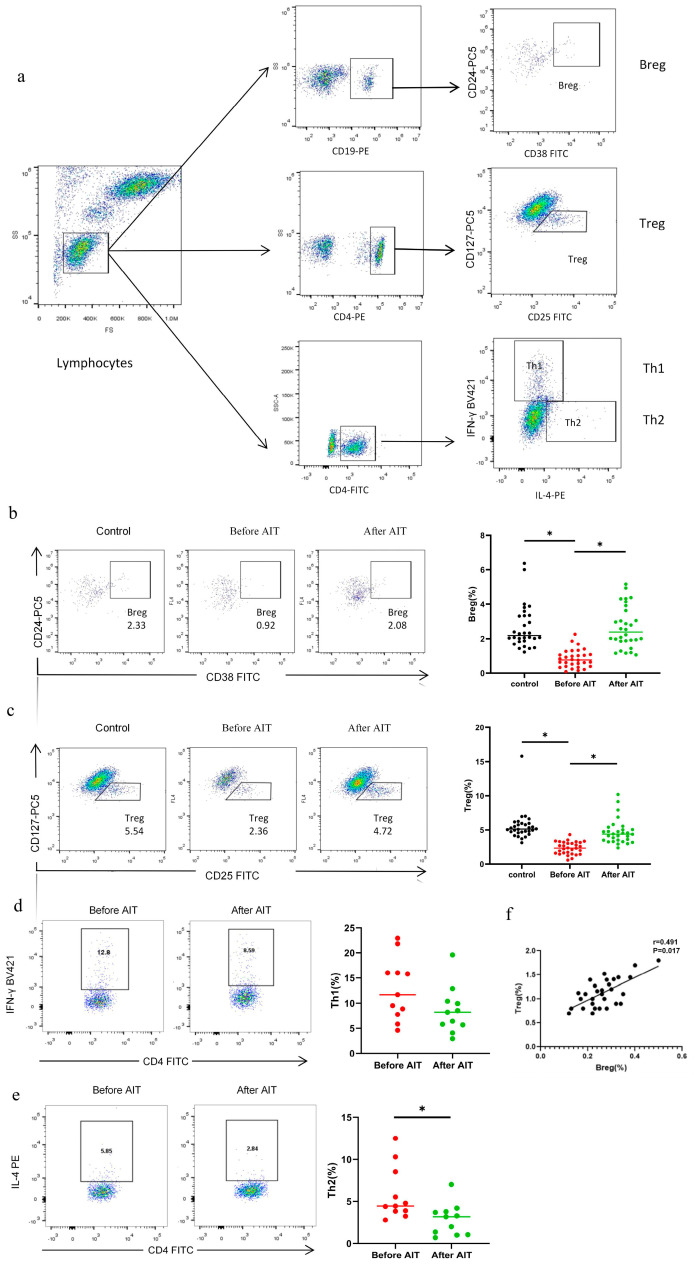
Comparison of peripheral blood Breg and Treg in patients with AR before and after AIT. (**a**) Flow cytometry gating strategies for Breg, Treg, Th1 and Th2. (**b**,**c**) The proportion of peripheral blood Bregs and Tregs in AR patients increased significantly after AIT. The number of Bregs and Tregs is positively correlated. (**d**,**e**) Comparison of CD4^+^IFN^-^γ^+^Th1 cells and CD4^+^IL-4^+^Th2 cell in peripheral blood of AR patients before and after AIT. Th1 cells decreased slightly after treatment, with no statistical difference. Th2 cells decreased significantly after treatment. (**f**) Correlation between Bregs and Tregs in peripheral blood of patients with AR. *: Significantly different from the control group (*p* < 0.05, respectively).

Our results showed that, compared to the healthy control group, AR patients had significantly reduced levels of IL-10 and TGF-β in peripheral blood (Figure 1d,e), as well as decreased numbers of Breg and Treg cells in both peripheral blood and serum. After AIT, the levels of Breg and Treg in peripheral blood and serum of AR patients returned to normal levels (Figure 2b,c), and the Th1/Th2 ratio increased significantly (Figure 2d,e). Additionally, there was a positive linear correlation between CD19^+^CD24^hi^CD38^hi^Breg and CD4^+^CD25^+^CD127^-^Treg in peripheral blood (Figure 2f).

### 3.4. Preparation of Dust Mite Allergen Gene Der p1-DC Vaccine

**Figure 3:**
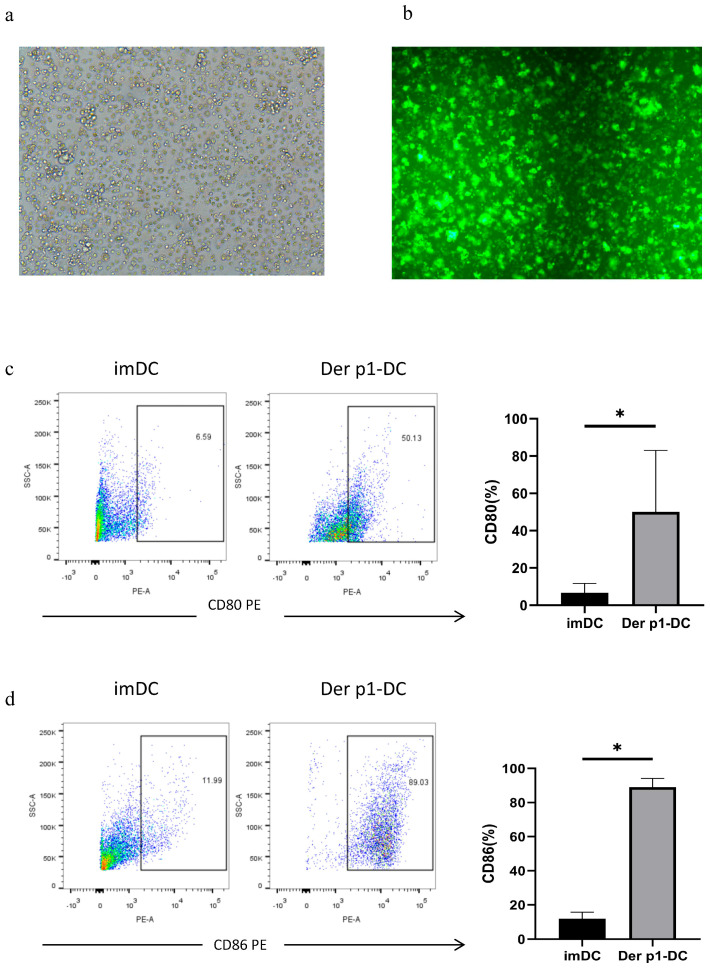
Preparation of Der p1-DC. (**a**,**b**) Expression by Der p1 modified DCs in fluorescence and bright light microscopy showed the successful infection of Lenti-Derp1-GFP into DCs (original magnification: ×10). (**c**,**d**) Flow cytometry was used to detect the DC maturation markers CD80 and CD86. It was found that immature dendritic cells (imDC) expressed a small amount of maturation markers. In Der p1-DC, an increase in the expression of CD80 and CD86 was observed. *: Significantly different from the control group (*p* < 0.05, respectively).

The transfected DCs were observed under a fluorescence microscope (Figure 3a,b). Flow cytometry was used to detect DC maturation markers CD80 and CD86. Immature dendritic cells (imDCs) expressed low levels of these maturation markers (Figure 3c,d). In Der p1-DCs, increased expression of CD80 and CD86 was observed (Figure 3c,d). These results suggest that the Der p1-DC vaccine was successfully prepared and had a high degree of maturity, allowing it to further exert immune effects.

### 3.5. DC Vaccines Induce Breg Differentiation In Vitro and Enhance Immune Inhibition Through IL-10

**Figure 4:**
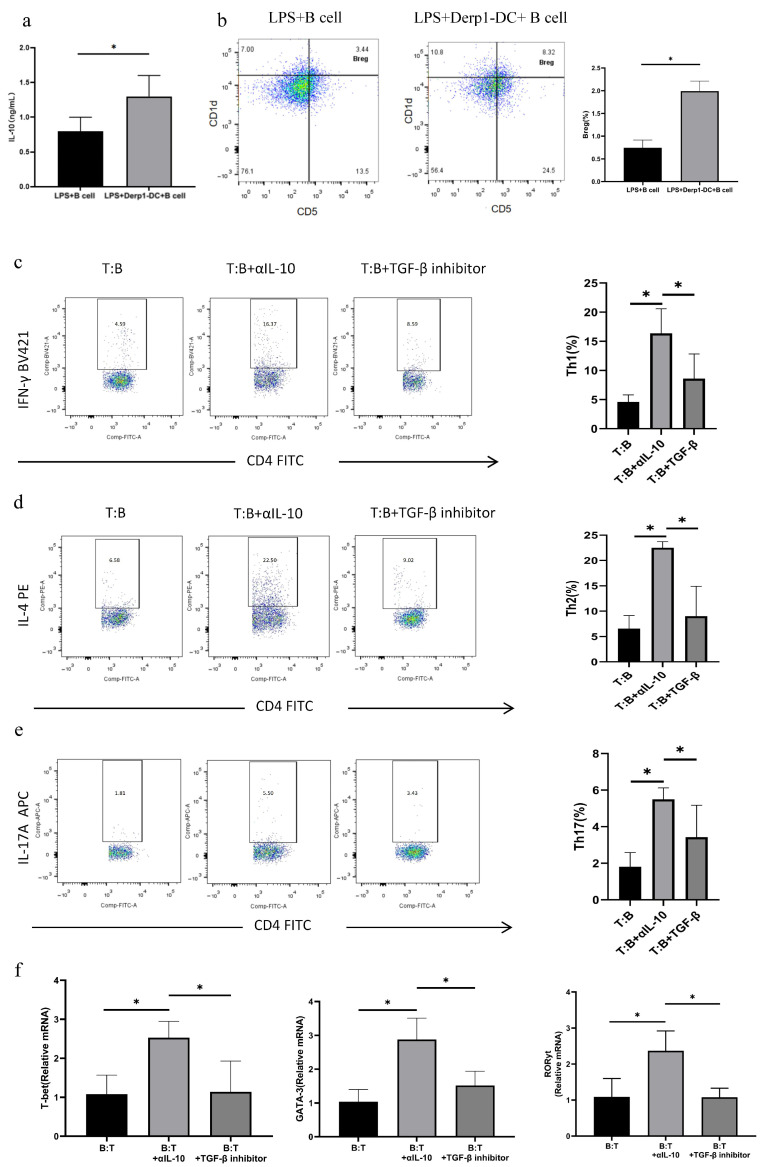
Effect of Bregs induced by Der p1-DC on Th cell differentiation. (**a**) The expression level of IL-10 in the supernatant was detected by ELISA. It was found that the level of IL-10 in the supernatant of the co-culture of Der p1-DC and B cells was significantly higher than that in the supernatant of B cells cultured alone. (**b**) Flow cytometry was used to detect the level of Breg cells. It was found that after B cells were induced by the combination of Der p1-DC and LPS, the level of CD19^+^CD5^+^CD1d^+^Breg cells was significantly higher than that induced by LPS alone. (**c**–**e**) After co-culture of Bregs induced by Der p1-DC and CD4^+^CD25^-^T cells, the number of Th1, Th2 and Th17 cells in IL-10 blocked group was significantly higher than that in non-blocked group; The proportions of Th1, Th2 and Th17 cells in TGF blocking group were slightly higher than that in non-blocking group, and there were no significant difference between groups. (**f**) After co-culture of Bregs induced by Der p1-DC and CD4^+^CD25^-^T cells, the mRNA expressions of t-Bet, GATA3, RORγt in IL-10 blocking group were significantly higher than that in non-blocking group, and there was no significant difference between TGF-β blocking group and non-blocking group. *: Significantly different from the control group (*p* < 0.05, respectively).

Mouse splenic B cells were obtained by magnetic bead separation. After LPS stimulation and differentiation, B cells were co-cultured with Der p1-DC vaccines. ELISA was used to detect the expression level of IL-10 in the supernatant. The results showed that the level of IL-10 in the supernatant of Der p1-DC and B cell co-cultures was significantly higher than that in B cell cultures alone (Figure 4a). Flow cytometry was used to detect Breg levels, and it was found that the level of CD19+CD5+CD1d+Breg after induction with Der p1-DC combined with LPS was significantly higher than that induced by LPS alone, suggesting that Der p1-DC can enhance the induction of Breg differentiation (Figure 4b). Mouse CD19+CD5+CD1d+Breg and splenic CD4^+^CD25^-^T cells were further sorted. Breg cells were co-cultured with CD4^+^CD25^-^T cells. The results showed that the proportions of Th1, Th2, and Th17 cells were higher in the IL-10 blockade group than in the non-blockade group, while there was no significant difference in the proportions of these cells between the TGF-β blockade group and the non-blockade group (Figure 4c–e). Similarly, RT-PCR results showed that the mRNA expression levels of T-bet, GATA3, and RORγt were significantly higher in the IL-10 blockade group than in the non-blockade group, while there was no significant difference in the mRNA expression levels of these genes between the TGF-β blockade group and the non-blockade group (Figure 4f).

### 3.6. Der p1-DC Treatment Improves Nasal Symptoms in AR Mice

**Figure 5:**
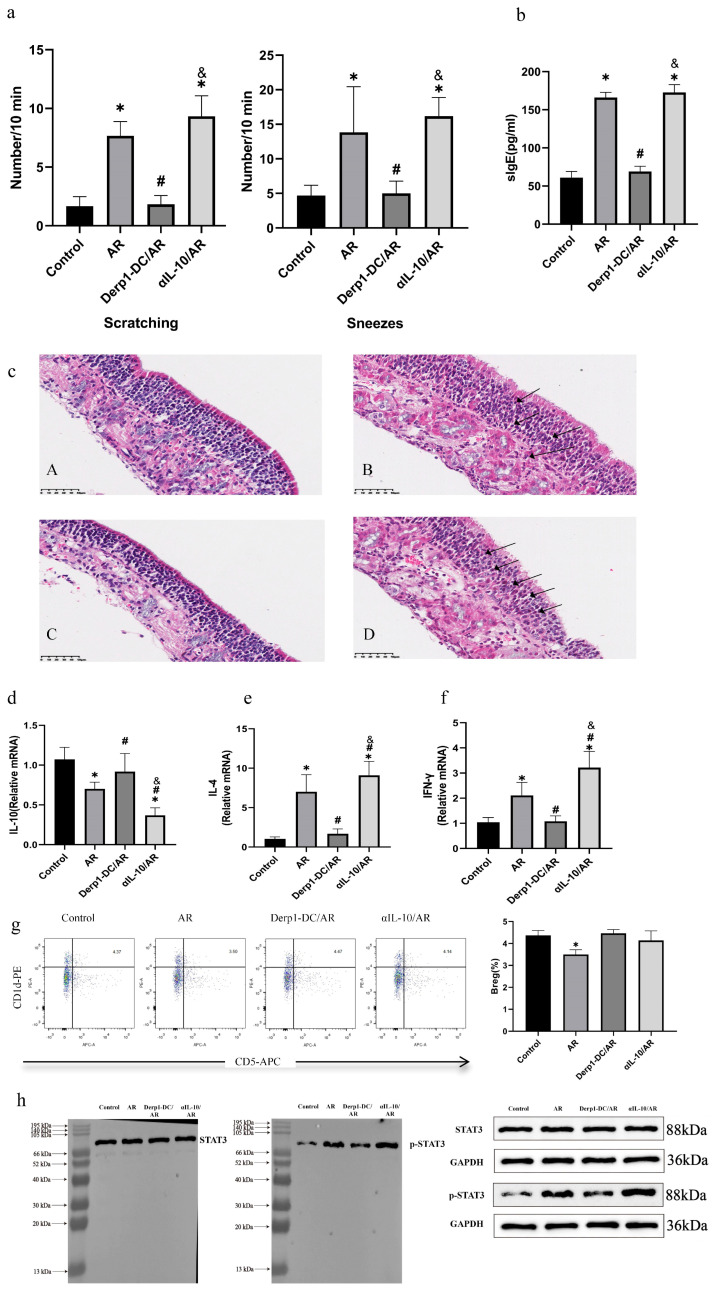
Comparison of mice before and after treatment with Der p1-DC. (**a**) After 4 weeks of treatment, compared with the AR group, the number of nose scratching and sneezing episodes in the Der p1-DC/AR group of mice was significantly reduced. However, there was no significant change in the IL-10 antibody blocked/AR group. * indicates *p* < 0.05 compared to the control group; # indicates *p* < 0.05 compared to the AR group; & indicates *p* < 0.05 compared to the Der p1-DC/AR group. (**b**) Compared with the control group, the levels of serum specific IgE in the AR group and the IL-10 antibody blocked/AR group increased significantly. The level of serum specific IgE in the Der p1-DC/AR group decreased significantly. (**c**) Histological observation showed no significant eosinophil infiltration in the control group (**A**). In contrast, substantial eosinophil infiltration was observed in the nasal mucosal stromal tissue of the AR group (**B**) and the IL-10 antibody blockade/AR group (**D**), while the Der p1-DC/AR group (**C**) showed less eosinophil infiltration. Arrows indicate eosinophils (H&E staining, ×400). (**d**–**f**) Compared with the control group, the level of IL-10 in the nasal mucosa of the AR group and the IL-10 antibody blocked/AR group decreased significantly. Moreover, the expression of IL-10 mRNA in the IL-10 antibody-blocked/AR group was lower than that in the AR group. The level of IL-10 mRNA in the Derp1-DC/AR group was significantly higher than that in the AR group and the IL-10 antibody blocked/AR group. The expressions of IL-4 and IFN-γ mRNA in the nasal mucosa showed opposite trends among the various groups. * indicates *p* < 0.05 compared to the control group; # indicates *p* < 0.05 compared to the AR group; & indicates *p* < 0.05 compared to the Der p1-DC/AR group. (**g**) The proportion of Breg cells in the AR group was significantly lower than that in the control group, while the proportions of Breg cells in the Derp1-DC/AR group and the IL-10 antibody-blocked/A group were significantly higher than that in the AR group. The proportion of Breg cells in the IL-10 antibody-blocked/AR group was slightly lower than that in the Derp1-DC/AR group, but there was no significant difference. (**h**) Western blotting was used to evaluate p-STAT3/STAT3 in the spleens of mice. The results showed that compared with the control group, p-STAT3 in the AR group and the IL-10 antibody blocked/AR group was significantly increased. Moreover, the ratio of p-STAT3/STAT3 in the IL-10 antibody-blocked/AR group was higher than that in the AR group. The ratio of p-STAT3/STAT3 in the Derp1-DC/AR group was lower than that in the control group.

Finally, we investigated the role of Breg induced by Der p1-DC in the formation of immune tolerance in AR mice. The Der p1-DC/AR group received intraperitoneal injections of Der p1-DC for treatment, while the IL-10 antibody blocked/AR group, AR group, and control group received PBS instead (Figure 5). Four weeks after treatment, it was found that compared to the AR group, the number of nasal scratching and sneezing in the Der p1-DC group was significantly reduced, while there was no significant change in the IL-10 antibody blockade/AR group (*n* = 6 per group) (Figure 5a).

### 3.7. Serum Specific IgE Levels and Histological Observation of Nasal Mucosa

Compared to the control group, the serum specific IgE levels were significantly increased in the AR group and IL-10 antibody blockade/AR group. In the Der p1-DC group, the serum specific IgE levels were significantly reduced (Figure 5b). Histological observation showed that there was a large infiltration of eosinophils in the interstitial tissue of the nasal mucosa in the AR group and IL-10 antibody blockade/AR group, while there was less eosinophil infiltration in the Der p1-DC group (Figure 5c).

### 3.8. Expression of IL-4, IFN-γ, and IL-10 mRNA in Nasal Mucosa

Compared to the control group, the expression of IL-10 in the nasal mucosa was significantly reduced in the AR group and IL-10 antibody blockade/AR group. Additionally, the expression of IL-10 mRNA in the IL-10 antibody blockade/AR group was lower than that in the AR group. The level of IL-10 mRNA in the Der p1-DC/AR group was significantly higher than that in the AR group and IL-10 antibody blockade/AR group. The expression of IL-4 and IFN-γ mRNA in the nasal mucosa showed an opposite trend among the groups. These results indicate that Der p1-DC treatment in AR mice can upregulate the level of IL-10 in the nasal mucosa and inhibit the expression of IL-4 and IFN-γ. After IL-10 antibody blockade, the expression of IL-4 and IFN-γ in the nasal mucosa of AR mice further increased, suggesting that Der p1-DC can inhibit the expression of IL-4 and IFN-γ by upregulating the expression of IL-10 (*n* = 6 per group) (Figure 5d–f).

### 3.9. Proportion of Bregs in Mouse Spleen and Expression of STAT3 and Phosphorylated STAT3 in the Spleen

The proportion of Breg cells in the AR group was significantly lower than that in the control group, while the proportion of Breg cells in the Der p1-DC/AR group and the IL-10 antibody-blocked/AR group was significantly higher than that in the AR group. The proportion of Breg cells in the IL-10 antibody-blocked/AR group was slightly lower than that in the Der p1-DC/AR group, but there was no significant difference (Figure 5g). The p-STAT3/STAT3 ratio in mouse spleen was assessed by Western blotting, and the results showed that compared with the control group, p-STAT3 was significantly increased in both the AR group and the IL-10 antibody-blocked/AR group. Furthermore, the p-STAT3/STAT3 ratio in the IL-10 antibody-blocked/AR group was higher than that in the AR group. The p-STAT3/STAT3 ratio in the Der p1-DC/AR group was lower than that in the control group (*n* = 3 per group) (Figure 5h).

## 4. Discussion

Our research has initially revealed that AIT significantly ameliorates the symptoms of AR and concurrently augments the proportions of peripheral blood CD19^+^CD24^hi^CD38^hi^Breg and CD4^+^CD25^+^CD127^-^Treg, with a positive correlation being manifested between them. These outcomes indicate that Breg exerts a crucial function in AIT for AR, and the combined assay of CD19^+^CD24^hi^CD38^hi^Breg and CD4^+^CD25^+^CD127^-^Treg can act as a potential biomarker for prognostic evaluation in AIT.

The induction and maintenance of immunological tolerance are central mechanisms for restoring immune balance and counteracting allergic inflammation [14]. AIT primarily functions by inducing the generation and activation of Treg and Breg to repair impaired allergic tolerance [10,15,16]. Previous studies have consistently shown that airway allergic diseases are associated with reduced numbers and functional defects of Breg and Treg. Our study confirms lower proportions of CD19^+^CD24^hi^CD38^hi^Breg in seasonal AR patients compared to healthy controls [17]. Following 6 months of AIT, AR patients exhibited significant relief of nasal symptoms, restoration of the peripheral blood Th1/Th2 balance, and marked increases in Breg and Treg proportions with positive correlation, indicating quantitative or functional deficiencies of Breg in AR patients and AIT’s role in inducing immunological tolerance via upregulating Breg and Treg levels.

IL-10 is a key cytokine through which Breg modulate immune responses and suppress excessive inflammation. Its functions include inhibiting Th1 and Th17 cell responses, promoting Treg proliferation and upregulating FOXP3 expression, suppressing antigen presentation and pro-inflammatory cytokine production by DC, monocytes, and macrophages, maintaining invariant natural killer T (iNKT) cell numbers and function, and inducing IgG4 production [18]. In a house dust mite-sensitized murine model, adoptively transferred CD9^+^ Breg cells secrete IL-10 via the mitogen-activated protein kinase pathway to suppress Th2/Th17 inflammation, restore the Treg/Th2 ratio, and induce apoptosis of CD3^+^CD4^+^CD25^−^ effector T cells, thereby reestablishing immune balance in lung tissue [19]. Our study found significantly lower peripheral blood IL-10 levels in AR patients compared to healthy controls, with levels returning to normal after AIT. This suggests that AIT may promote high IL-10 expression in Breg, Treg, and other cells to inhibit Th2-type inflammation and restore Treg/Th2 balance.

Current AIT faces challenges such as limited efficacy, long treatment duration, and poor patient compliance. DC vaccines have emerged as a promising alternative in respiratory allergic diseases like asthma [20]. Our prior study developed a Der p1-DC via lentiviral transfection, demonstrating its ability to relieve allergic symptoms and upregulate Treg in AR mice, but its effect on Breg remained unclear [21,22]. In vitro experiments showed that Der p1-DC promotes B cell differentiation into Breg, with differentiated Breg primarily inhibiting Th1, Th2, and Th17 cells via IL-10 rather than TGF-β. Co-culture of magnetically sorted mouse splenic B cells with Der p1-DC significantly increased IL-10 levels in culture supernatants. Flow cytometry revealed higher proportions of CD19^+^CD5^+^CD1d^+^ Breg induced by Der p1-DC combined with LPS compared to LPS alone, confirming Der p1-DC’s enhanced capacity to induce Breg differentiation.

Breg primarily suppress Th2-mediated inflammation by secreting cytokines like IL-10 and TGF-β, restoring Th1/Th2 balance in AR patients [23]. Additionally, AR patients exhibit increased Th17 cells, whose IL-17 secretion recruits neutrophils and drives inflammatory responses [24]. Co-culture experiments with mouse CD19^+^CD5^+^CD1d^+^ Breg and CD4^+^CD25^−^ T cells showed significant suppression of Th1, Th2, and Th17 differentiation by Breg. Blocking IL-10 significantly attenuated this suppression, while TGF-β blockade had no effect, suggesting that Der p1-DC-induced Breg primarily exert immunosuppression via IL-10 secretion.

Our prior research has indicated that Der p1-DC is capable of facilitating the differentiation of Treg cells within AR mice, consequently repressing the Th2 response and ameliorating the symptoms of AR allergic reactions [21,22]. It serves as an efficacious therapeutic approach for inducing immune tolerance. Thus, in order to further probe into the immunoregulatory function of Der p1-DC in AR, we examined the mechanism of Der p1-DC in treating AR by regulating the expression of IL-10, the phosphorylation of STAT3, and the differentiation of Breg in a mouse model. The outcomes demonstrated that Der p1-DC could relieve the nasal symptoms of AR mice and decrease the level of serum specific IgE. This study also uncovered that during the process of allergic inflammation, Der p1-DC could restrain the expression of IL-4 and suppress the allergic inflammation response via the Breg/IL-10/STAT3 signaling pathway.

Currently, it is widely recognized that IL-10 functions by binding to its receptor, which subsequently activates JAK1 and TYK2. This activation leads to the suppression of the release of pro-inflammatory factors [25]. In the context of this research, within the Der p1-DC/AR group of mice, an elevation in the proportion of Breg in the spleen was observed, accompanied by an upregulation of IL-10 levels and a reduction in IL-4 levels in the nasal mucosa. In the AR mice of the IL-10 antibody blocking group, the IL-10 level in the nasal mucosa declined even further, while the IL-4 level increased. These findings suggest that treatment with modified DC has the potential to induce immune tolerance in AR mice by enhancing the levels of Breg and its secreted IL-10. Considering that the experimental subjects are mice and their peripheral blood volume is relatively limited, it is primarily utilized for detecting alterations in relevant immune parameters such as specific IgE in the serum. The spleen, being the largest lymphoid organ in the body and a major site for immune responses, is rich in lymphocytes. Thus, we opted to examine the expression of p-STAT3 and STAT3 in the mouse spleen to gain a deeper understanding of the mechanism underlying Der p1 vaccine immunotherapy. When compared to the control group, a significant increase in p-STAT3 was noted in both the AR group and the IL-10 antibody blocking/AR group. Moreover, the p-STAT3/STAT3 ratio in the αIL-10/AR group was higher than that in the AR group, while the P-STAT3/STAT3 ratio in the Der p1-DC/AR group was lower. It is noteworthy that our study found that in the Der p1-DC/AR group, an increase in IL-10 levels was accompanied by a decrease in the p-STAT3/STAT3 ratio. This appears to contradict the classical understanding that the IL-10 signaling pathway directly induces STAT3 phosphorylation. We speculate that this seemingly paradoxical result may stem from our observation representing the integrated output of a complex network of immune cell interactions. The high levels of IL-10, likely derived from induced regulatory cells such as Bregs, may shape an overall anti-inflammatory microenvironment. This environment potently suppresses the STAT3 phosphorylation primarily driven by pro-inflammatory factors like IL-6 and GM-CSF, which occurs chiefly in effector T cells (e.g., Th17) or inflammatory antigen-presenting cells. The inhibitory effect on this pro-inflammatory STAT3 signaling may outweigh the transient activation of STAT3 by IL-10 in its direct target cells (e.g., macrophages). Consequently, this leads to a net decrease in the p-STAT3/STAT3 ratio at the bulk protein level. This highlights the complexity of IL-10’s role in allergic immunoregulation: it functions not only by directly activating STAT3 within target cells but also, more importantly, by systemically altering the cytokine milieu to indirectly modulate STAT3 activity across different cell populations, ultimately resulting in a net immunosuppressive effect. Future studies should employ techniques such as sorting specific cell subsets for phospho-signaling analysis to more precisely dissect the specific impact of IL-10 on STAT3 signaling in different cell types. Our research outcomes collectively demonstrate that Der p1-DC can suppress STAT3 phosphorylation through the high expression of IL-10, thereby exerting an immunosuppressive effect.

In this study, we observed in the mouse model that intraperitoneal injection of Derp1-DC enables sustained expression of the house dust mite allergen Derp1 in vivo. This strategy induces immune tolerance through mechanisms such as promoting the differentiation of Breg, thereby providing solid preclinical evidence for a novel approach to allergen-specific immunotherapy for allergic rhinitis and revealing its potential therapeutic targets. The Derp1-DC vaccine strategy holds the promise of reducing the frequency of dosing for patients, which is a major factor contributing to poor patient compliance. However, we also note that in clinical research, DC vaccines remain primarily used in oncology. Translating DC vaccine therapy for allergic diseases into clinical practice still presents significant challenges. Several limitations must be addressed before clinical application, including the safety profile of Derp1-DC, the optimal concentration for eliciting the desired immune response, the appropriate dosing frequency, and the identification of biomarkers to monitor treatment efficacy.

## 5. Conclusions

This study elucidates that AIT restores immune balance in AR by upregulating Breg and Treg and promoting IL-10 secretion, while Der p1-DC vaccine enhances immunological tolerance via the Breg/IL-10/STAT3 pathway. Future research should validate the clinical value of combined Breg/Treg detection as AIT biomarkers and explore optimized protocols for DC vaccine-AIT combination therapy, providing new directions for precision medicine in AR.

## Figures and Tables

**Table 1:** Baseline Comparison of the Study Subjects.

	AR Group	Control Group
N	30	30
Age	27.27 ± 16.17	27.67 ± 16.22
Sex		
Male	16 (53.33%)	16 (53.33%)
Female	14 (46.67%)	14 (46.67%)
BMI (kg/m^2^)	23.34 ± 8.55	21.97 ± 4.30
Past medical history		
Yes	30 (100.0%)	2 (6.67%)
No	0	28 (93.33%)

## Data Availability

The raw data supporting the conclusions of this article will be made available by the authors on request.

## Abbreviations

The following abbreviations are used in this manuscript:

AR	Allergic Rhinitis
Breg	Regulatory B Cell
DC	Dendritic Cell
AIT	Allergen Immunotherapy
IgE	Immunoglobulin E
Treg	Regulatory T Cell
Der p1	Dermatophagoides Pteronyssinus 1
ELISA	Enzyme-Linked Immunosorbent Assay
imDC	Immature Dendritic Cell
iNKT	Natural Killer T Cell
